# Capecitabine plus bevacizumab versus capecitabine in maintenance treatment for untreated characterised KRAS exon 2 wild-type metastatic colorectal cancer: a retrospective analysis in Chinese postmenopausal women

**DOI:** 10.1186/s12876-018-0916-6

**Published:** 2019-01-25

**Authors:** Jinsong Su, Jiajie Lai, Ruikun Yang, Bo Xu, Ying Zhu, Mingdong Zhao, Chen Yang, Guanzhao Liang

**Affiliations:** 1 Department of Colorectal and Anal Surgery, The First Affiliated Hospital of Zhengzhou University, Jianshe East Road No.1, Erqi District, Zhengzhou, 450052 Henan China; 2 Department of Gynaecology and obstetrics, The First Affiliated Hospital, Sun Yat-sen University, Huangpu East Road No. 183, Huangpu District, Guangzhou, 510700 China; 3 Department of Pediatrics, The First Affiliated Hospital, Sun Yat-sen University, Huangpu East Road No. 183, Huangpu District, Guangzhou, 510700 China; 4 Department of thoracic surgery, The First Affiliated Hospital, Sun Yat-sen University, Huangpu East Road No. 183, Huangpu District, Guangzhou, 510700 China; 5 Department of Radiology, The First Affiliated Hospital, Sun Yat-sen University, Huangpu East Road No. 183, Huangpu District, Guangzhou, 510700 China; 6 Department of Orthopaedics, Jinshan Hospital, Fudan University, Longhang Road No. 1508, Jinshan District, Shanghai, 201508 China; 7 Department of Physical Examination, The First Affiliated Hospital, Sun Yat-sen University, Huangpu East Road No. 183, Huangpu District, Guangzhou, 510700 China; 8 Emergency Department, The First Affiliated Hospital, Sun Yat-sen University, Huangpu East Road No. 183, Huangpu District, Guangzhou, 510700 China

**Keywords:** Capecitabine, Bevacizumab, Colorectal cancer, Progression-free survival, Overall survival

## Abstract

**Background:**

Capecitabine plus bevacizumab (CAP-B) maintenance treatment after 6 cycles of capecitabine, oxaliplatin, and bevacizumab (CAPOXB) has demonstrated clinical activity and failure to compromise quality of life in patients with metastatic colorectal cancer (MCC) in a previous phase 3 CAIRO3 study. The objective of this study is to evaluate the efficacy and safety of CAP-B versus CAP in maintenance treatment after 6-cycle CAPOXB induction therapy in Chinese postmenopausal women with untreated characterised KRAS exon 2 wild-type MCC.

**Methods:**

During 2012–2016**,** prospectively maintained databases were reviewed to evaluate cohorts with untreated characterised KRAS exon 2 wild-type MCC and stable disease or better after 6-cycle CAPOXB induction treatment. After induction treatment, all patients received either CAP-B or capecitabine (CAP) as maintenance treatment. Median progression-free survival (mPFS) and median overall survival (mOS) were the primary endpoints. Safety was the secondary endpoint.

**Results:**

A total of 263 women with untreated characterised KRAS exon 2 wild-type MCC and stable disease or better after 6-cycle CAPOXB induction treatment were included for the evaluation of efficacy and safety (CAP-B-treated cohort, *n* = 130 and CAP-treated cohort, *n* = 133). The mPFS was 11.5 months (95% confidence interval [CI], 5.6–17.4) and 9.2 months (95% CI, 3.6–14.8) for the CAP-B-treated and CAP-treated cohorts, respectively (HR 0.54, 95% CI 0.32~0.85; *P* = 0.013). The mOS was 16.2 months (95% CI, 11.4–18.7) and 12.4 months (95% CI, 10.6–15.5) for the CAP-B- and CAP-treated cohorts, respectively (HR 0.72, 95% CI 0.51~0.94; *P* = 0.022). The CAP-B-treated cohort experienced significantly more grade 3 or 4 diarrhoea (*P* < 0.001) than the CAP-treated cohort.

**Conclusions:**

CAP-B maintenance treatment after 6-cycle CAPOX-B in Chinese postmenopausal women with untreated KRAS exon 2 wild-type MCC is poorer tolerated but has a more modest, if any, benefit compared with that of CAP maintenance treatment.

## Background

The estimated incidence of stage 1–3 colorectal cancer in China for 2017 is 100,000 patients, of which almost 70% did develop metastatic colorectal cancer (MCC), which is partially related to common delays in diagnosis [1–3]. Despite the lack of multi-centre, open-label, double-arm trials that compared the efficacy and safety of different therapeutic regimens in patients with MCC, 4 prospective studies comparing the efficacy and safety of bevacizumab(B) and capecitabine (CAP) had indicated a safe and effective regimen for the treatment of patients with late-stage colorectal cancer [3–6]. However, evidence from recent studies suggests that MCC carrying extended KRAS exon 2 mutations predicted for the lack of activity of anti-epidermal growth factor receptor (EGFR) therapies and resulted in restriction to the use of anti-EGFR monoclonal antibodies, making these drugs suitable for the so-called “super” wild-type patients only [7–9]. Approximately 70% patients with KRAS exon 2 wild-type(wt) MCC tend to have a poor prognosis, with a median overall survival (mOS) of 4–8 months and a median progression-free survival (mPFS) of 3–9 months [10–12].

KRAS exon 2 wt MCC has historically demonstrated restricted response to intervention, and prospective trials with small samples have presented poor outcomes [10, 11]. Despite recent developments in targeted therapies, experience with these agents in KRAS exon 2 wt MCC is still inadequate [12]. Previous retrospective studies have either excluded or lacked definite details of the inclusion of KRAS exon 2 wt MCC [13, 14]. At the time of study development, bevacizumab, a recombinant humanised monoclonal immunoglobulin G antibody, was considered a favourable antiangiogenic agent and was approved by the US Food and Drug Administration(UFDA) for the first-line treatment of KRAS exon 2 wt MCC [15]. The combination regimen of bevacizumab and CAP in MCC has been reviewed extensively [16]. In previous retrospective studies, the addition of bevacizumab was observed to significantly improve the patient’s mPFS and mOS, with 5–7 months and 5–18 months for bevacizumab in comparison to 3–6 months and 4–12 months for placebo, respectively [17]. The results of a prior phase 3 CAIRO3 trial demonstrated that MCC patients with stable disease or better after 6-cycle induction treatment with capecitabine, oxaliplatin, and bevazicumab (CAPOXB) had a significant benefit from CAP-B maintenance treatment compared to observation [18]. Although the trial was not premeditated to detect a difference in mOS between groups, an absolute mOS benefit of 3.5 months was detected, which was not statistically significant (HR0.89, 95% CI 0.73–1.07). In addition, in the phase 3 CAIRO3 trial, the analysis of this type of patients with untreated characterised KRAS exon 2 wt MCC is disregarded. A multi-center, single-arm, phase II trial (CCOG-0801) has been conducted to investigate targeted therapy alone, where either bevacizumab was unavailable or the toxicity profile of bevacizumab had been considered adverse [19]. Their study included 47 patients from 2008 to 2010 with untreated characterised KRAS exon 2 wt MCC, and patients underwent 6-cycle CAPOXB induction therapy, followed by CAP-B or CAP maintenance treatment. The CAP maintenance treatment group described 2-year clinical outcomes that were encouraging, with mPFS of 54% and mOS of 63%. Nevertheless, with a median follow-up of 30 months, adverse events had occurred in 9 (19.1%) cases, which raises questions about whether CAP maintenance treatment was acceptable in the absence of bevacizumab, regardless of the cost-effectiveness of CAP-B. Furthermore, hormone-based therapy, a potential MCC preventive intervention, may influence carcinogenesis through cellular pathways involving the KRAS oncogene in postmenopausal women. Nevertheless, most existing literature tend to ignore hormones as an interference factor, which is likely to lead to a weakening of the power to draw conclusions.

The purpose of this study was to compare the safety and efficacy of CAP and CAP-B as maintenance treatment after 6-cycle-CAPOXB induction treatment for Chinese postmenopausal women with untreated characterised KRAS exon 2 wt MCC even after progression with these drugs during the treatment.

## Methods

### Study design and patient eligibility

A retrospective study was conducted on Chinese postmenopausal women with untreated characterised KRAS exon 2 wt MCC at 3 tertiary medical institutions from January 2012 to December 2016. MCC was defined by the International Classification of Disease Clinical Modification 10th edition ICD-10 code (C.18–C.20). For these patients, the analysis results of EGFR next-generation sequencing of the RAS/BRAF/PI3KCA genes were available. The study cohort included 412 patients diagnosed with untreated characterised KRAS exon 2 wt MCC. Diagnosis of metastasis was based on contrast-enhanced computed tomography (CT), contrast-enhanced magnetic resonance imaging (MRI), or positron emission tomography–computed tomography (PET-CT) scan, and these patients had received initial 6-cycle CAPOXB induction therapy, followed by either capecitabine plus bevacizumab (CAP-B) or capecitabine (CAP) maintenance treatment. Inclusion criteria: patients age range 60–80 years, with histologically proven metastatic colorectal adenocarcinoma; patients with KRAS exon 2 mutation detected by high-resolution melting analysis; unresectable metastasis; an Eastern Collaborative Oncology Group (ECOG) performance score of 0–2 and a life expectancy of 2 years, excluding their colorectal cancer diagnosis; at least one measurable lesion evaluated according to the Response Evaluation Criteria in Solid Tumours (RECIST) version 1.1 [20] by using either CT, MRI or PET-CT; adequate haematologic, liver, and renal function (neutrophil granulocytes ≥1.5 × 10^9^/L; thrombocytes ≥75 × 10^9^/L; creatinine clearance ≥30 mL/min; total bilirubin concentration ≤ 2 times the upper normal limit; liver transaminase or alkaline phosphatase ≤3 times the upper limit of normal; and concentrations). Exclusion criteria: patients with failure to undergo rigid proctoscopy; another primary cancer within 5 years; non-healed or planned surgery; uncontrollable underlying diseases; brain metastasis; thrombotic episode within 6 months; clinically significant cardiovascular or cerebrovascular diseases; clinical obstruction requiring a temporary diverting ostomy or endorectal stent to maintain bowel patency; modification, discontinuation or interruption of CAP-B or CAP regimen; blood system or liver diseases; previous chemoradiotherapy; uncontrolled diabetes mellitus or hypertension; severe infectious; cognitive dysfunction; mental illness; incomplete medical records; and refusal to participate.

### Definitions of the descriptive variables

Patients with untreated characterised KRAS exon 2 wt MCC was defined as that patients with characterised KRAS exon 2 wt MCC had failed to undergo radiotherapy, chemotherapy, or surgical resection of colorectal cancer before 6-cycle CAPOXB induction therapy. PFS was calculated from the onset of maintenance until the date of disease progression or death from any cause. OS was alculated from the onset of maintenance until the date of death for any cause. The tumour cell content of each sample included was assessed on haematoxylin–eosin stained slides by an experienced pathologist. EGFR mutation analysis was performed by using the next-generation sequencing method of the RAS/BRAF/PI3KCA genes, as described [2, 21]. Symptom assessment were performed every 3 months for the entire study period. Metastatic recurrence was confirmed mainly on the basis of imaging data. The rating rules for adverse events was based on Common Terminology Criteria for Adverse Events (NCI-CTCAE) version 4.0. Medical therapy was performed in line with institutional guidelines. Assessment of response or progression was on the basis of either MRI or endorectal ultrasound. Temporary diverting ostomy was mainly based on the general situation of patients.

### Study design and treatment

A retrospective, multi-centre study was performed, in which eligible patients received 6-cycle CAPOX-B induction therapy, as described by Nakayama et al. [19]. CAP-B maintenance therapy (intravenous B 7.5 mg/kg once a day and intravenous CAP 1000 mg/m^2^ twice daily) or CAP maintenance therapy (intravenous CAP 1000 mg/m^2^ twice daily) was performed for patients with stable or better following 6-cycle CAPOX-B induction therapy. The primary endpoints were mPFS and mOS. The secondary endpoint was safety.

### Statistical analysis

The X-square test or Mann–Whitney U-test was used to compare categorical data. Continuous data were expressed as mean ± SD. A two-sample Student’s t test was applied to analyse differences in continuous data. The primary endpoints were compared using the log-rank test and were assessed by the Kaplan-Meier methods to estimate the mean and median with 95% CI. Cox hazard model were executed to assess the effect of CAP-B and CAP on PFS and OS. Statistical programming and analyses were produced using SPSS software, version 24.0 (IBM, Armonk, New York, USA). All statistical tests were two-sided. Differences were considered statistically significant at *P* < 0.05.

## Results

### Comparison of baseline data

**Fig. 1:**
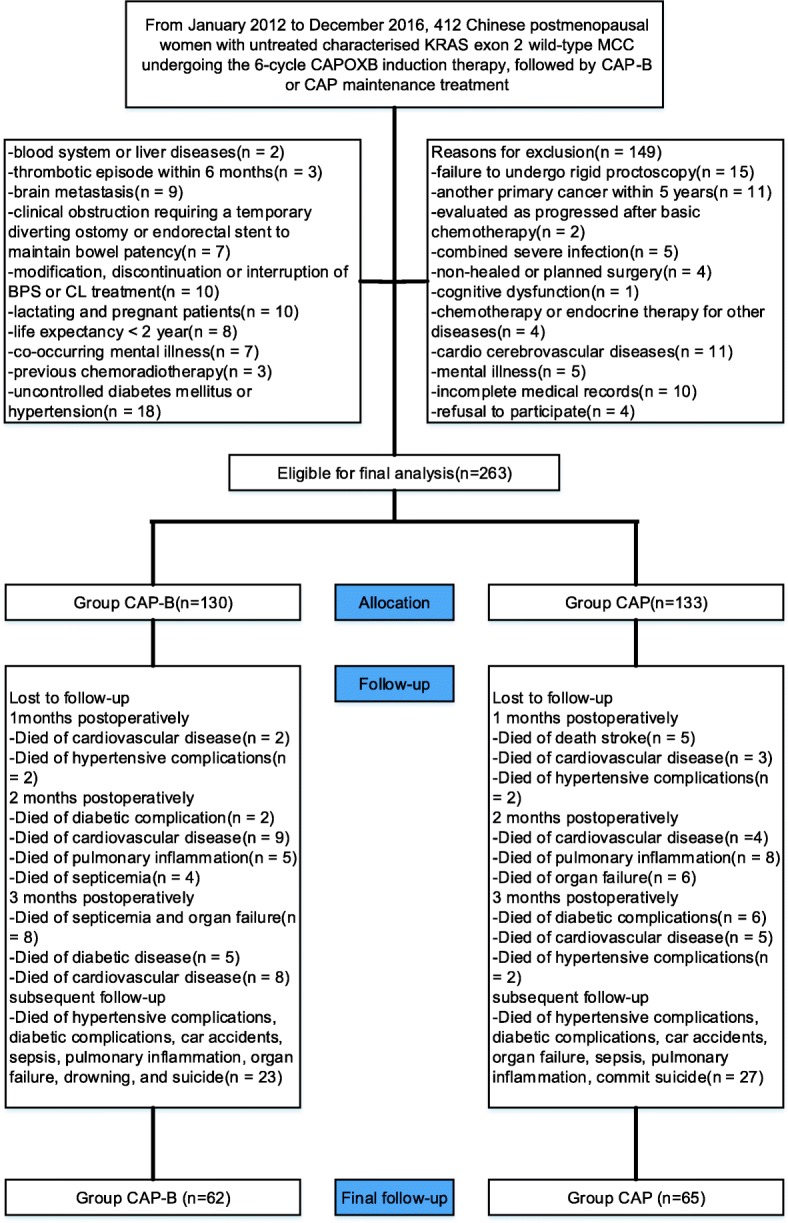
Flow diagram demonstrating methods for identification of studies to retrospectively evaluate the efficacy and safety of CAP-B versus CAP in maintenance treatment after 6-cycle CAPOXB induction therapy in Chinese postmenopausal women with untreated characterised KRAS exon 2 wild-type MCC

From January 2012 to December 2016, the cohort comprised 263 postmenopausal women (CAP-B-treated cohort *n* = 130, mean age 68.6 years [SD 8.43] and CAP-treated cohort *n* = 133, 69.1 years [SD 8.27]) for study eligibility (Fig. 1). The median follow-up time of all cases included in the study was 15.2 months (range 3.1–39.4 months). The mean duration of the study at the primary analysis cut-off date was 25.2 months (IQR 6.0–42.3) for patients on CAP-B and 24.7 months (IQR 3.4–46.0) for patients on CAP, and the last follow-up time was January 2018. Characteristics of the 263 cases from which all relevant information was available are presented in Tables 1 and 2. No significant difference was detected in baseline data between groups.

**Table 1:** Patient demographics between groups

Variable	CAP-B (*n* = 130)	CAP (*n* = 133)	*P*-value
Age at onset (years)	68.6 ± 8.43	69.1 ± 8.27	0.247^*a^
Site of primary tumour			0.806^*b^
Caecum to transverse colon	58	60	
Splenic flexure to rectum	47	52	
Multiple sites	25	21	
Duration of treatment (mos)	24.2 ± 18.14	24.7 ± 21.33	0.129*^a^
Performance Status(ECOG)			0.514^*b^
0	76	83	
1	54	50	
Number of metastatic sites			0.586^*b^
1	57	62	
> 1	43	40	
unknown	30	31	
Best response to induction treatment			0.612^*b^
Stable disease	52	59	
Partial response	36	35	
Complete response	11	13	
No change	21	18	
Progression	10	8	
Time from induction treatment to start of maintenance treatment			0.385^*b^
≤ 1 mos	102	110	
> 1 mos	28	23	
Primary site			0.639^*b^
Colon	52	57	
Rectum	78	76	

^*^No statistically significant values. ^a^Analysed using an Independent-Samples t-test; ^b^Analysed using the Mann-Whitney test. *CAP-B* capecitabine plus bevacizumab, *CAP* capecitabine, *ECOG* Eastern Collaborative Oncology Group

**Table 2:** Comparison of the result of the treatment of Asian patients with untreated characterised KRAS exon 2 wt MCC between groups at the final follow-up

Variable	CAP-B (n = 130)	CAP (n = 133)	*P*-value
Deaths	64	85	0.016*^a^
Recurrence	7	18	0.024*^a^
Metastatic brain/leptomeningeal tumours	11	18	0.189^a^
> 3 metastases^b^	13	24	0.061^a^

^*^Statistically significant values. ^a^Analysed using the chi-square test. ^b^including the brain, bone, lung, liver, and lymph nodes. *CAP-B* capecitabine plus bevacizumab, *CAP* capecitabine, *MCC* metastatic colorectal cancer

### Comparison of efficacy

**Fig. 2:**
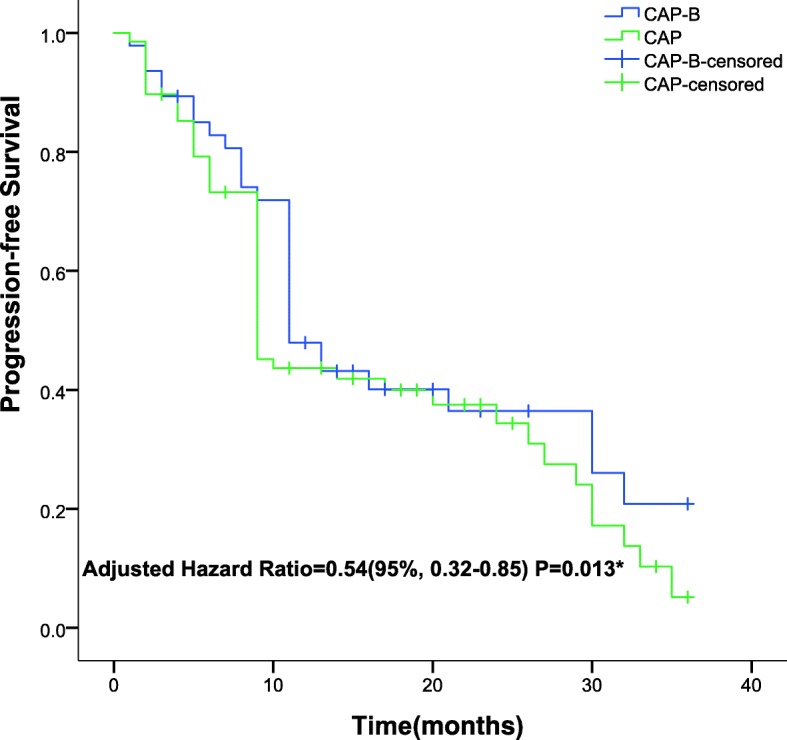
Kaplan–Meier Curves for progression-free survival**.** The median progression-free survival was respectively 9.2 months (range, 3.6–14.8 months) in the CAP group; the median progression-free survival was 11.5 months (range, 5.6–17.4 months) in the CAP-B group. Statistically significant difference was detected in the progression-free survival between groups. *Hazard ratio was calculated using a Cox proportional-hazards model, with the type of age, site of primary tumour, number of metastatic sites, and performance status as covariates and CAP/CAP-B therapy as time-dependent factor. With respect to the progression-free survival, results of a log-rank test, *P* = 0.013

**Fig. 3:**
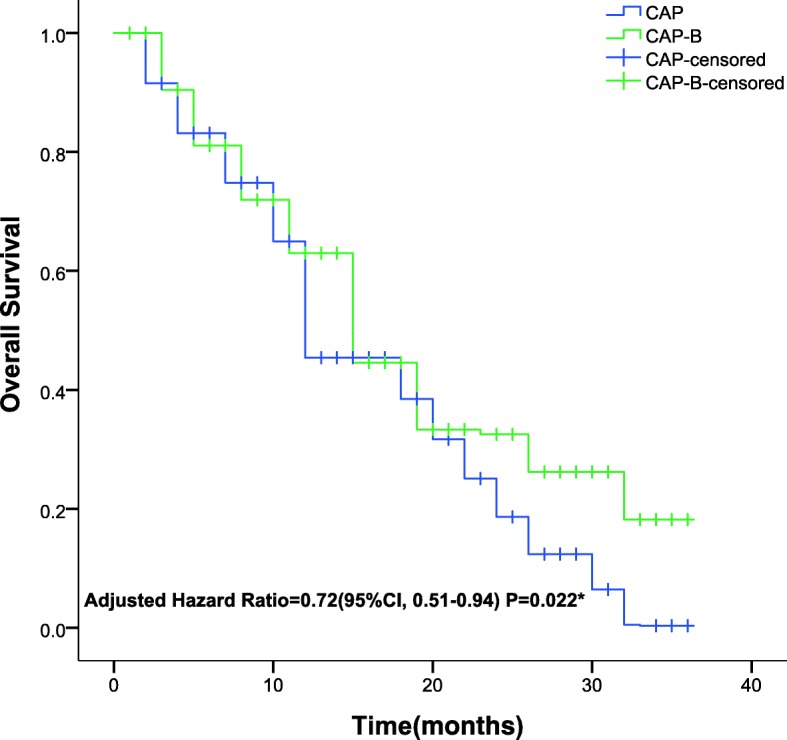
Kaplan–Meier Curves for overall survival. The median overall survival was 16.2 months (range, 9.2–23.2 months) in the CAP-B group; the median overall survival was 12.4 months (range, 5.9–18.9 months) in the CAP group. Significant difference was detected in the overall survival between groups. *Hazard ratio was calculated using a Cox proportional-hazards model, with the type of age, site of primary tumour, number of metastatic sites, and performance status as covariates and CAP/CAP-B therapy as time-dependent factor. With respect to the overall survival, results of a log-rank test, *P* = 0.022

The mPFS, one of the primary endpoints, was 11.5 months (95% CI, 5.6–17.4 months) for the CAP-B-treated group and 9.2 months (95% CI, 3.6–14.8) for the CAP-treated group. The mOS was 16.2 months (95% CI, 11.4–18.7) for the CAP-B-treated cohort and 12.4 months (95% CI, 10.6–15.5) for the CAP-treated cohort, as presented in Table 3. Significant differences in the mPFS (0.54, 95% CI 0.32~0.85; *P* = 0.013) (Fig. 2) and mOS (0.72, 95% CI 0.51~0.94; *P* = 0.022) (Fig. 3) were detected between the CAP-treated and CAP-B-treated cohorts, respectively. The 2-group cohorts received no surgical treatment for primary or metastatic lesions.

**Table 3:** Comparison of the progression-free survival and overall survival between groups at the final follow-up

Variable	CAP-B (*n* = 130)	CAP (*n* = 133)	*P*- value
Median progression-free survival (mos)	11.5 ± 5.93	9.2 ± 7.61	0.013*^a^
Median overall survival (mos)	16.2 ± 7.02	12.4 ± 6.52	0.022*^a^
Progression-free survival rate	29.2%	16.5%	0.014*^b^
Overall survival rate	50.8%	36.1%	0.016*^b^

^*^Statistically significant values. ^a^Analysed using the independent-samples t-test. ^b^Analysed using the Kaplan-Meier method. *CAP-B* capecitabine plus bevacizumab, *CAP* capecitabine

### Adverse events

The incidence of major drug-related adverse events is presented in Table 4. The frequencies of haematological and non-haematological adverse events exceeding grade 3 were 13.8 and 6.9% for the CAP-B- and CAP-treated groups, respectively, for the complete treatment period. Peripheral sensory neuropathy occurred in 36 cases (13.7%), including 8 cases (3.0%) with 3-grade peripheral sensory neuropathy, of which 5 occurred at the end of the 3rd month of the CAP-B therapy and 3 occurred at the end of 4th month of the CAP therapy. The drug-related toxicities observed occurred at a moderately constant rate of cases throughout the overall treatment period; nevertheless, treatment discontinuation occurred in 17 cases (11 [8.5%] for CAP-B and 6 [4.5%] for CAP) that were excluded from the study for myocardial ischaemia, apparently owing to drug-related coronary vasospasm, and 5 cases (3.8%) that failed to complete the maintenance therapy as a result of bevacizumab-related toxicities (2 for 2-grade mucositis and 3 for 3-grade dehydration and 3-grade diarrhoea. Of 263 cases, 29 (11.0%) completing the initial CAPOX-B induction therapy had a requirement of reduction in the CAP-B or CAP dose: 22 (16.9%) required a reduced CAP-B dose, 7 (5.3%) required a reduced CAP dose. Diarrhoea, the most frequent 4-grade toxicity eliminating lymphopenia, occurred in 10.8 and 3%, followed by rash in 13.3 and 6.4%, in the CAP- and CAP-B-treated cohorts, respectively(*P* = 0.013).

**Table 4:** Comparison of the incidence of major drug-related adverse events between groups at the final follow-up

Adverse events	CAP-B (*n* = 130)	CAP (*n* = 133)	*P*-value
Haematological and non-haematological events	18(13.8%)	9(6.9%)	0.059^a^
Peripheral sensory neuropathy	21(16.2%)	15(11.3%)	0.250^a^
Myocardial ischaemia	11(8.5%)	6(4.5%)	0.193^a^
4-grade diarrhoea	14(10.8%)	4(3.0%)	0.013*^a^

^*^Statistically significant values. ^a^Analysed using the Chi-square test. *CAP-B* capecitabine plus bevacizumab, *CAP* capecitabine

## Discussion

The present study followed Chinese postmenopausal women with untreated KRAS exon 2 wt MCC for a mean of 2 years, and the most important finding was that CAP-B is a feasible maintenance treatment for these patients after 6-cycle CAPOX-B induction treatment compared with CAP. The superiority of CAP-B over CAP after 6-cycle CAPOX-B in Chinese postmenopausal women with untreated KRAS exon 2 wt MCC remains a matter of debate, which precludes any recommendations.

In most patients, in daily practice, KRAS mutational status is evaluated in samples originating from primary intestinal lesions at the time of diagnostic colonoscopy [9, 12]. The rationale for the application of anti-EGFR monoclonal antibodies in KRAS exon 2 wt MCC cases depended on the appropriate concordance of mutational status between primary and metastatic tumours, as presented in previous literature [22, 23]. Nevertheless, noteworthy differences in the incidence of KRAS exon 2 mutations among tumour locations have been reviewed [8, 9, 24]. The superiority of CAP-B over CAP remains controversial, which precludes any recommendations [2, 6, 7]. A growing but still very limited body of literature comparing the clinical efficacy of CAP-B and CAP in the management of Chinese postmenopausal women with untreated KRAS exon 2 wt MCC after 6-cycle CAPOX-B induction treatment demonstrated comparable outcomes [10]. Chen et al. [25] noticed a longer mPFS in postmenopausal women receiving CAP-B treatment than those recieving CAP treatment at a mean follow-up of 2 years. Our finding further expounded the significant differences in the mPFS between groups but were inconsistent with several prior retrospective reports that showed no significant differences in the mPFS [14, 22]. Furthermore, a prospective study by Yamaguchi et al.[26]comprising 31 cases with untreated KRAS exon 2 wt MCC receiving CAP-B or CAP treatment after 6-cycle CAPOX-B induction treatment confirmed no significant difference in the mPFS.

As using chemotherapy alone in the current treatment only has a modest, if any, benefit, we wanted to evaluate whether CAP-B or CAP as maintenance treatment after 6-cycle CAPOX-B induction treatment could improve mPFS and/or mOS in untreated KRAS exon 2 wt MCC [27]. Only a few 3 phase II trials comparing CAP-B with CAP in similar regimens showed no improvement in mPFS or mOS [1, 26, 27]. Comparing with prior trials using the identical strategy with bevacizumab, the last study reported by Gervais et al.[18]failed to obtain benefit, although CAP-B, which had been investigated in a small population of 27 cases, had an extraordinary mOS of 2 years.

This study undoubtedly showed that Chinese postmenopausal women with untreated KRAS exon 2 wt MCC managed in the CAP-B or CAP setting have almost indistinguishable 2-year PFS and OS when treated using neoadjuvant chemotherapy first, followed by chemoradiation and then surgery [16]. Moreover, compared to no addition, the addition of bevacizumab produced statistically significant benefit [15]. The CAP-B or CAP maintenance treatment has been espoused by previous investigations [1, 15, 28]. Based on the above facts, CAP-B or CAP has become the wide-reaching favoured approach for those women with untreated KRAS exon 2 wt MCC [1]. The application of CAPOX-B administration rather than other regimens was based predominantly on a US Intergroup study indicating outstanding outcomes with CAP therapy [1, 2]. However, the CAP strategy was observed to be relevant to outcomes inferior to those with the CAP-B strategy in Chinese postmenopausal women with untreated MCC. A previous trial evaluating CAP as a maintenance treatment failed to reach its primary endpoint of increasing the mPFS from 2 to 4 months [18]. Hence, CAP showed no distinct benefit for the cohort. In addition, the end-points, as mentioned above, tended to be inferior to the outcomes from previous meta-analyses with bevacizumab [29, 30]. The current study consequently seemed to support the treatment concept that combining bevacizumab with CAP in maintenance treatment was superior to CAP alone. Nevertheless, comparison of the results from different maintenance treatments was challenging [15]. The attributes of research object were difficult to achieve relative consistency. Thus, the study only demonstrated that combining bevacizumab with CAP in these cases previously treated with 6-cycle CAPOX-B induction treatment tended to result in a benefit.

Despite the small sample of the study and the drawback of the retrospective study, the strength and consistency of the CAP-B treatment results corroborated the findings from previous multi-centre studies [31, 32]. The current therapeutic regimen was clearly different from previous neoadjuvant chemotherapy regimens reported by several Asian researchers [33, 34]. Given the positive mPFS and mOS of our results in the CAP-B setting in untreated KRAS exon 2 wt MCC, we have an optimistic opinion that the positive results were attributable to bevacizumab. However, despite the documented benefits of bevacizumab in the management of MCC, rare adverse reactions have been reported, i.e., thromboembolic events, diarrhea, wound healing abnormalities, irreversible leuco-encephalopathy syndrome [29–31]. In addition, maintenance treatment with CAP-B does not appear to be cost-effective [9].

This study should be interpreted in light of important limitations. First, the nature of the retrospective study with all the problems inherent with this methodology limits the level of confidence of our conclusion, and it is possible that every potential confounding variable, such as underlying diseases, failed to be addressed in our analyses. Second, limited sample size may have introduced bias. Nevertheless, the focus of our study is to assess an area that has not been studied extensively in the literature. Third, our analysis has the lack of generalisability because our study population included only postmenopausal women with KRAS exon 2 wild-type MCC. Fourth, every attempt was made to adjust for all potential confounders, but other unmeasured factors may also be relevant.

## Conclusions

In summary, the study confirms that CAP-B maintenance treatment after 6-cycle CAPOX-B in Chinese postmenopausal women with untreated KRAS exon 2 wt MCC is poorly tolerated but has a more modest, if any, benefit compared with that of CAP maintenance treatment. Further evidence-based prospective evaluation of the long-term efficacy and safety of CAP or CAP-B in these patients with untreated KRAS exon 2 wt MCC should be expanded using randomised controlled trials.

## Abbreviations

B: Bevacizumab
CAP: Capecitabine
CAP-B: Capecitabine plus bevacizumab
CAPOXB: Capecitabine, oxaliplatin, and bevacizumab
CI: Confidence interval
CT: Computed tomography
ECOG: Eastern collaborative oncology group
EGFR: Epidermal growth factor receptor
HR: Hazard ratio
IQR: Interquartile range
MCC: Metastatic colorectal cancer
mOS: Median overall survival
mPFS: Median progression-free survival
MRI: Nuclear magnetic resonance imaging
PET-CT: Positron emission tomography–computed tomography
RECIST: Response evaluation criteria in solid tumours
UFDA: US Food and Drug Administration
wt: Wild-type
